# The impact of total knee arthroplasty on functional outcomes and mental health: A prospective study

**DOI:** 10.1002/jeo2.70314

**Published:** 2025-07-18

**Authors:** Bassem Haddad, Tasneem N. Alhosanie, Noor Yousef, Ali Kanaan, Leen A. Mazahreh, Khuzama Mohammad, Aya Al‐Zurgan, Leen Al‐Zghoul, Tala M. Mesmar, Fahad Alabhoul, Abdel Rahman Abuawad, Feras Abuhajleh, Mohammad Hamdan

**Affiliations:** ^1^ Division of Orthopedics, School of Medicine, Department of Special Surgery The University of Jordan Amman Jordan; ^2^ Software Engineering Department King Hussein School of Computing Sciences Princess Sumaya University for Technology Amman Jordan; ^3^ Research Assistant, School of Medicine The University of Jordan Amman Jordan; ^4^ School of Medicine The University of Jordan Amman Jordan; ^5^ Jordanian Royal Medical Services Amman Jordan; ^6^ Ministry of Health Kuwait; ^7^ King Hussein Cancer Center Amman Jordan

**Keywords:** anxiety, depression, knee function, knee osteoarthritis, primary total knee arthroplasty

## Abstract

**Purpose:**

Knee osteoarthritis (OA) is a leading cause of disability and chronic pain; total knee arthroplasty (TKA) is the effective treatment in the end stages of knee OA after failure of conservative management. This study has focused on the effects of primary TKA on knee function, levels of depression, and anxiety.

**Methods:**

The study population is a cohort of 100 patients who underwent primary TKA in a tertiary center in Jordan. Oxford Knee Score (OKS), Patient Health Questionnaire‐9 and Generalized Anxiety Disorder‐7 score assessments were performed preoperatively and three months postoperatively.

**Results:**

The results showed a dramatic improvement in knee function, with the mean OKS increasing from 17.9 preoperatively to 38.4 postoperatively (*p* < 0.001). Moreover, the percentage of patients who reported no or mild depression increased from 62% to 91%, and those who reported minimal or mild anxiety also increased from 76% to 95%. A multivariate linear regression showed that age, osteoporosis, and smoking were independently associated with the anxiety and depression score changes. These represent not only pain improvement but also a dual benefit in the field of mental health following TKA.

**Conclusion:**

Primary TKA showed significant improvement in patients' depression and anxiety, along with the improvement of their knee functional scores. Discussing psychological factors with patients preoperatively might be important along with the functional outcome in making the surgical decision.

**Level of Evidence:**

Level II, prospective cohort study.

**Abbreviations**
GAD‐7: Generalized Anxiety Disorder‐7
OA: knee osteoarthritis
OKS: Oxford Knee Score
PHQ‐9: Patient Health Questionnaire‐9
TKA: total knee arthroplasty
VAS: Visual Analogue Scale

## INTRODUCTION

Knee osteoarthritis (OA) is a major cause of pain and disability globally [24]. Conservative measures like patient education, exercise, weight loss and analgesics are the first‐line therapy, but should they fail, a total knee arthroplasty (TKA) is an effective modality that restores the functionality of the knee joint and alleviates chronic pain [9, 54].

Physicians are increasingly advised to holistically approach patients by addressing their physical and mental well‐being as part of comprehensive care. Both depression and anxiety are part of patients' overall health‐related quality of life (QoL) [11] and should be addressed in an effort to reduce any patient dissatisfaction [29]. One study showed that greater disability and decreased quality of life are factors contributing to a higher prevalence of anxiety among the elderly [35]. In the case of knee OA, higher rates of pain [3], healthcare costs [20], complication risks [2], analgesics [51, 61] and opioid use [26] have all been linked to higher preoperative anxiety and depression. Moreover, patients with end‐stage knee OA show higher rates of anxiety and depression when compared with age‐matched controls [37, 50].

The impact of TKA on QoL is well described in the literature. According to the National Joint Registry, 84% of patients who underwent TKA report that they are satisfied or at least ‘good’ [45]. Neuprez et al. lauded the advantages of knee replacement obtained in the long term, as continued functional benefits and reduced pain have been reported 5 years after the replacement [46]. Similarly, West et al. demonstrated that patients with TKA had satisfactory outcomes with substantially improved postoperative Knee Society Scores (KSS) compared to preoperative scores [63]. However, it should be noted that up to 44% of patients reported some form of pain following surgery [5, 49].

TKA's effects also extend to mental well‐being. In the same paper, West et al. showed that QoL deteriorated among patients with severe knee OA due to reported depression and anxiety [63]. The pain relief and alleviation of functional and physical symptoms naturally lead to an improvement in mental well‐being [55]. Marked improvement in postoperative knee scores is associated with a significant reduction of depressive and anxiety symptoms among patients at 6 months [22] and 1 year [21]. Hence, TKA leads to better mental health outcomes [39].

Both mental and physical well‐being can be quantified using validated tools. The Patient Health Questionnaire‐9 (PHQ‐9) and the Generalized Anxiety Disorder 7 questionnaire (GAD7) are proven tools to screen for depressive and anxiety symptoms, respectively. The KSS and the Oxford Knee Score (OKS) are knee OA‐specific tools. The OKS is particularly effective as it considers both functional status and pain when comparing pre‐and post‐operative scores [13].

Total knee arthroplasty (TKA)‐related dissatisfaction has been well‐documented and linked to a variety of conditions [33]. Naturally, there are also disadvantages to TKA, such as variable outcomes among patients.

During the first six months post‐TKA, 11.8% and 41.2% of patients experience major and minor complications, respectively. Common major complications include reoperation (2.5%) and TKA‐related readmission (6.0%) for reasons like surgical site infection, deep vein thrombosis, or manipulation under anaesthesia. The most common minor complications included joint stiffness 18.5%, unexpected pain 9.8%, swelling 15.6%, and paraesthesia 15.6% [25]. Interestingly, patients in a Dutch qualitative study repeatedly mentioned these common minor complications as causes of their dissatisfaction and negative mental health.

While TKA did relieve joint pain, some complain that it was replaced by a new distinct type of pain. Other complaints include pain flares during physical activity, tight/stiff knee and incomplete recovery of mobility and function that continued to affect their mental state. Finally, there was an uncomfortable period of 'getting used' to the prosthesis that generally persisted for around 7 months and, in some cases, up to 2 years. The same paper described that this patient dissatisfaction also negatively affected knee surgeons and other medical staff as they were disgruntled with the suboptimal outcomes [56].

This study aims to measure the short‐term impact of TKA on the mental well‐being of patients with primary knee OA in Jordan in correlation with the improvement of their physical well‐being. This is in an effort to better contextualise the patients' postsurgical course of anxiety and depression and to help physicians develop better, more patient‐centred treatment plans.

## METHODS

### Design and participants

**Figure 1:**
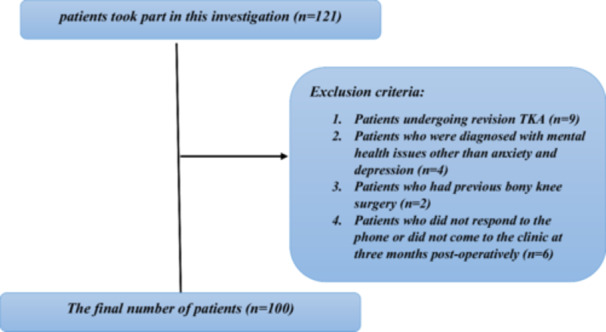
Inclusion and exclusion criteria flowchart.

Patients undergoing primary TKA in one tertiary hospital in Jordan (Jordan University Hospital) from January 2023 to January 2024 were included in the study. Patients consented to answering the questionnaires. These were filled pref‐operatively and 3 months post‐operatively. 121 consecutive patients were recruited, however, only 100 patients were followed up, completing all the questionnaires. Patients undergoing revision TKA, those diagnosed to have mental health issues other than anxiety and depression, those who had previous bony knee surgery, and patients who did not respond to our team's phone calls or did not visit the clinic at three months post‐operatively were excluded. Figure 1 shows the Inclusion and exclusion criteria flowchart.

Participants' data were collected from patients who visited the orthopaedic clinics pre‐operatively and 3 months post‐operatively via face‐to‐face interviews. Patients whose data was not collected during their visit to the clinic at 3 months post‐operatively were contacted by phone. All participants filled out the PHQ‐9, VAS, GAD‐7, and OKS scores along with extra questions about medical conditions and demographic features.

### Screening instrument

#### OKS

Knee pain and function (daily living activities) were measured using the OKS, which served as the outcome measure and was translated into Arabic by Ahmad et al. [1]. Patients were regularly given this questionnaire before and after TKA [10, 43]. The 12 questions that compromise the OKS were evaluated on a 5‐point scale from 0 (severe) to 4 (none). The scores were added up to provide an overall score, with 0 being the lowest possible score and 48 being the highest possible score.

#### PHQ‐9

A validated Tunisian version of the PHQ‐9, for major depressive disorder, was used to assess depressive symptoms [29]. The PHQ‐9 assessed both somatic (a range of physical sensations that a depressed person interprets as unpleasant or concerning) and mental (emotions about themselves and mental health issues) symptoms of depression. A total of nine items on a 4‐point rating system were included, with 0 being 'not at all' and 3 representing 'nearly every day'. A total score between 0 and 27 was computed using the response options; scores of 9 or less reflected 'mild' or 'no depression', scores between 10 and 14 reflected 'moderate depression', scores between 15 and 19 reflected 'moderately severe depression', and scores between 20 and 27 reflected 'severe depression' [36].

#### GAD‐7

The GAD‐7 is a self‐reported anxiety severity assessment. The Arabic version that was translated by Terkawi et al. was used [57]. The seven items of the GAD‐7 quantify the severity of different GAD symptoms based on reported response categories that are given points. The total score, which is the sum of the scores on each of the seven items' scales, indicates the assessment. The total score of the seven components of GAD‐7 ranges from 0 to 21 with cut‐off points of 5, 10 and 15 for mild, moderate and severe anxiety respectively [53].

#### Visual Analogue Scale (VAS)

The VAS is a valid and dependable technique for measuring pain that may be used to assess both acute and chronic pain [6]. The scale length is 10 cm. 'The worst imaginable pain' is assessed as 10 points (10 cm scale) on the VAS, whereas 'no pain' is typically rated as 0 points [23]. The patient is instructed to draw a marking on the line that accurately represents his/her pain level.

### Statistical analysis

The study's data were analysed using the Statistical Package for the Social Sciences (SPSS) version 22. In this study, descriptive statistics, t‐test, and multiple linear regression analysis (stepwise approach) were employed.

## RESULTS

This study included one hundred patients. Females made up 84.0% of the total sample. Table 1 shows that most participants were married, aged between 60 and 70 years (with a mean of 67 years), and most were unemployed. A large number of participants had only completed their diplomas or less in education. All patients underwent TKA due to primary osteoarthritis of their knees. Diabetes and hypertension have been identified as the most frequent comorbidities among the study participants. The majority of the participants were non‐smokers.

**Table 1:** Patient's demographics.

Variable	Category	Frequency	Percentage
Gender	Male	16	16.0%
	Female	84	84.0%
Age	50–60	19	19.0%
	60–70	50	50.0%
	>70	31	31.0%
Marital status	Single	6	6.0%
	Married	62	62.0%
	Widowed	32	32.0%
Education status	No school	27	27.0%
	High school	22	22.0%
	Diploma	26	26.0%
	University degree	25	25.0%
Work	Yes	29	29.0%
	No	71	71.0%
Smoking	Yes	7	7.0%
	No	93	93.0%
Comorbidities	DM alone	3	3.0%
	HTN alone	29	29.0%
	HTN and DM	17	17.0%
	HTN and other disease	9	9.0%
	HTN and DM and other	6	6.0%
	Other comorbidities	9	9.0%
	No disease	27	27.0%
Osteoporosis	Yes	24	24.0%
	No	48	48.0%
	Not test	28	28.0%

Abbreviations: DM, diabetes mellitus; HTN, hypertension.

Table 2 shows that there were significant differences regarding depression and anxiety levels when comparing patients' scores three months postoperatively to preoperatively (*p* = 0.000). Three months after surgery, there was a considerable increase in the percentage of patients reporting no anxiety or depression. Pre‐ and 3‐month post‐operative OKS scores also showed significant (*p* = 0.001) differences. The same results apply to the preoperative and the 3‐month post‐operative VAS scores, which were also significant.

**Table 2:** PHQ‐9, GAD‐7, VAS and OKS differences among patients before and after TKA.

Variables	Pre‐TKA (mean ± SD)	Three months post‐TKA (mean ± SD)	*t*‐test	*p* value
PHQ‐9	2.31 ± 1.07	1.27 ± 0.67	8.56	0.000**
GAD‐7	1.77 ± 0.99	1.14 ± 0.47	5.88	0.000**
VAS	7.98 ± 2.37	2.12 ± 1.91	20.38	0.000**
OKS	1.48 ± 0.688	3.31 ± 0.917	−17.8	0.000**

Abbreviations: GAD‐7, Generalized Anxiety Disorder 7; OKS, Oxford Knee Score; PHQ‐9, Patient Health Questionnaire‐9; TKA, total knee arthroplasty; VAS, Visual Analogue Scale.

**Figure 2:**
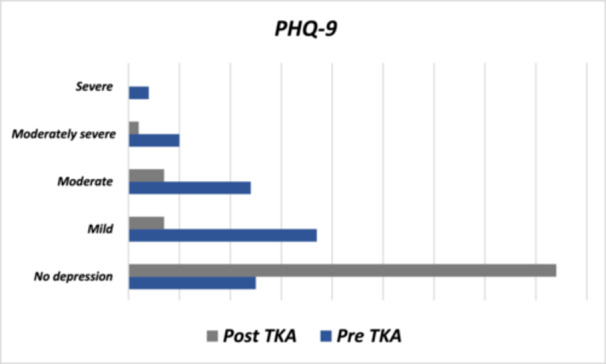
Depression pre‐ and post‐TKA. PHQ‐9, Patient Health Questionnaire‐9; TKA, total knee arthroplasty.

**Figure 3:**
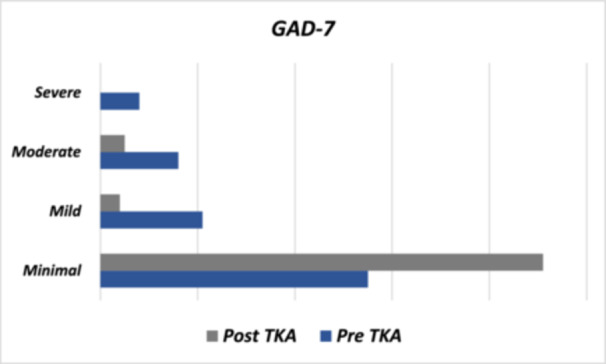
Anxiety pre‐ and post‐TKA. GAD‐7, Generalized Anxiety Disorder 7; TKA, total knee arthroplasty.

**Figure 4:**
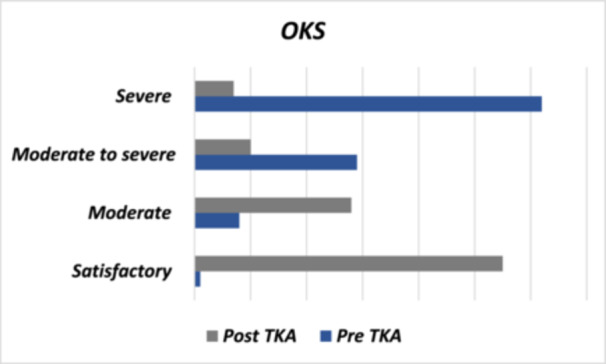
Oxford Knee Score (OKS) pre‐ and post‐total knee arthroplasty (TKA).

Figure 2 shows that 62.0% of patients before TKA reported having no or mild depression, and this percentage increased to 91.0% 3 months after surgery. Similarly, Figure 3 illustrates that 76.0% of patients reported having minimal or mild anxiety before surgery, and this percentage increased to 95.0% 3 months after the procedure. Regarding OKS, patients with severe and moderate to severe scores preoperatively represented 91.0%, which decreased to 17.0% 3 months later (Figure 4). Lastly, the average VAS score before TKA was 7.98 ± 2.37, which decreased to 2.12 ± 1.91 after the procedure (Table 1).

Multivariate linear regression was used to determine the impact of various variables on changes in PHQ‐9, GAD‐7, VAS and OKS as outcome variables. We investigated several different models to choose the optimal model based on the AIC value. The model based on age and GAD‐7 explained 41.5% (39.0% adjusted) of the variation in the change in PHQ‐9 (*p* < 0.000), according to our findings after applying step AIC. A statistically significant correlation was seen between these factors and the change in PHQ‐9 levels (Table 3). However, when looking at GAD‐7 as an outcome variable, we found that 50.6% (46.3% adjusted) of the variation in the change in GAD‐7 can be explained by age, smoking, osteoporosis and PHQ1 (*p* < 0.000). Finally, for VAS scores as outcome variables, we found that the studied variables did not explain the change in the values of these variables (Table 4).

**Table 3:** Results of linear regression analysis for PHQ‐9.

Term	*t*‐Value	*p* value	Coef.	CI (95%)
Intercept	1.112	0.2688	0.903	−0.70 to 2.51
Age	2.048	0.043	0.025	0.00–0.04
GAD‐7 mild	Reference			
GAD‐7 minimal	−3.850	0.000	−0.862	−1.30 to −0.41
GAD‐7 moderate	1.619	0.108	0.457	−0.10 to 1.01
GAD‐7 severe	4.066	0.000	1.432	0.73–2.13

Abbreviations: CI, confidence interval; GAD‐7, Generalized Anxiety Disorder 7; PHQ‐9, Patient Health Questionnaire‐9.

**Table 4:** Results of linear regression analysis for GAD‐7.

Term	*t*‐Value	*p* value	Coef.	CI (95%)
Intercept	5.690	0.00	4.125	2.68–5.56
Age	−3.496	0.007	−0.036	−0.05 to −0.01
Smoker no	Reference			
Smoker yes	−1.830	0.07	−0.548	−1.14 to 0.04
Osteoporosis no	Reference			
Osteoporosis, not test	−0.610	0.54	−0.107	−0.45 to 0.24
Osteoporosis yes	−2.037	0.044	−0.381	−0.75 to −0.009
PHQ‐9 mild	Reference			
PHQ‐9 no depression	−2.099	0.038	−0.40	−0.78 to −0.021
PHQ‐9 moderate	2.182	0.031	0.42	0.03–0.80
PHQ‐9 moderate severe	5.539	0.000	1.46	0.93–1.98
PHQ‐9 severe	5.016	0.000	1.92	1.16–2.68

Abbreviations: CI, confidence interval; GAD‐7, Generalized Anxiety Disorder 7; PHQ‐9, Patient Health Questionnaire‐9.

Regarding the impact of certain levels of GAD‐7 on PHQ‐9 levels, the results indicated that patients with minimal and severe levels of GAD‐7 had a significant association when we compared it with mild GAD‐7 patients. Moreover, age had a positive association with PHQ‐9 level (*p* = 0.04). On the other hand, age and individuals diagnosed with osteoporosis compared to those without osteoporosis and those not diagnosed with depression compared with mild level based on the PHQ‐9 scale had a negative association with GAD‐7 score value (*p* = 0.007, 0.04, 0.03) respectively. In addition, we found that patients with moderate, moderate to severe, and severe depression scores compared to mild depression scores had a positive association with GAD‐7 value (*p* = 0.03, 0.00, 0.00) respectively.

Osteoporosis, comorbidities, and pre‐operative OKS scores were used as independent factors in a multivariate regression model to analyse their effect on the change in OKS, which served as the outcome variable. 46% (or 41% adjusted) of the variation in the change in OKS was explained by this model (*p* < 0.00) (Table 5). Table 5 shows a statistically significant correlation between all these three factors and the change in OKS. The largest adjusted effect size (*p* < 0.00) was seen in pre‐operative OKS, where a lower pre‐operative OKS was linked to a more significant change in OKS (improvement). Moreover, the patients with comorbidities and osteoporosis exhibited the least improvement in OKS and vice versa (*p* = 0.001 and *p* = 0.003, respectively). This is because both osteoporosis and comorbidity indicated positive relationships (Table 5).

**Table 5:** Results of linear regression analysis for OKS.

Term	*t*‐Value	*p* value	Coef.	CI (95%)
Intercept	2.92	0.00	18.04	5.80‐30.2
Diabetes	Reference			
Hypertension	3.11	0.00	18.74	6.77‐30.7
Hypertension and diabetes	2.80	0.00	17.87	5.20‐30.5
Hypertension and diabetes and other disease	1.94	0.05	13.92	−0.32‐28.1
Hypertension and other disease	0.75	0.45	4.97	−8.08‐18.0
Other disease	2.53	0.02	16.70	3.63‐29.7
No disease	2.89	0.00	17.49	5.50‐29.4
Osteoporosis No	Reference			
Osteoporosis, not test	2.17	0.03	5.33	0.47‐10.2
Osteoporosis Yes	0.27	0.78	0.70	−4.46‐5.87
Pre OKS	5.016	0.000	1.92	−1.12‐ −0.65

Abbreviations: CI, confidence interval; OKS, Oxford Knee Score.

## DISCUSSION

This study's findings provide insight into patients' prognosis and quality of life after TKA. Information regarding pain, mental health, and functionality both before and three months after TKA are provided by this study. The study's most significant conclusions were that, after TKA, pain, depression, and anxiety decreased and that, three months after TKA, the majority of patients showed improvement in their OKS.

Due to variations in follow‐up, the anxiety and depression scales used, and demographics, it is challenging to compare the degree of changes in anxiety and depression in the literature. For instance, the Hospital Anxiety and Depression Scale (HADS) has been used in several studies to determine the incidence of anxiety and depression after TKA.

According to Duivenvoorden et al. [12], 1 year after surgery, the incidence of anxiety and depression dropped from 20.3% and 22.7%, respectively, to 14.8% and 11.7%. Likewise, 1 year following surgery, the incidence of anxiety and depression decreased from 35% and 22% to 26% and 9%, respectively, according to Jones et al. [30]. In the mean Hospital Anxiety and Depression Scale at 6 months postoperative, other studies have found raw score improvements of about 6.5 points [7] and 2 points [22] and a median improvement of 4 points in the PHQ‐9 at 1 year postoperative [14]. In our study, the mean scores of depression and anxiety decreased from 2.3 to 1.2 and from 1.7 to 1.1, respectively.

Nonetheless, our results showed that symptoms of anxiety and depression improved in 75%–100% of patients who had moderate to severe preoperative anxiety and depression. At three months after surgery, the proportion of patients showing moderate‐to‐severe anxiety dropped from 24% to 5.0%, and the proportion of patients reporting moderate‐to‐severe and severe depression decreased from 14.0% to 2.0%. Our results align with the more extensive literature corpus [59], indicating that TKA reduces anxiety and depression symptoms in patients suffering even severe mental symptoms as early as three months after surgery.

Comparing the preoperative baseline in PHQ‐9 scores, our results showed that 62.0% of the total 100 patients showed no or mild depression (score of 9 or less). Visser et al. in the USA classified this as No Major Depressive Disorder in their 2018 study of 260 patients, and they showed that 226 patients or 86.9% had no preoperative MDD (207 of the No MDD group and 19 in the Gained MDD group) [62]. Two other US studies also utilised the PHQ‐9 score, although they studied TKA and total hip arthroplasty together. They showed that 76.7% (215/280) [15] and 77.5% (76/98) [58] had no or mild preoperative depression per PHQ‐9. Therefore, we see our baseline depression values as comparable to other literature that utilised the same questionnaire.

Numerous studies have not used the PHQ‐9 or the GAD7 tools, but rather the HADS, but an attempt to contrast scores can still be made. Again, our results show that 76% showed no or mild preoperative depression on PHQ‐9, and 61% showed no or mild preoperative anxiety on GAD7. Taking a cut‐off point of < 8 on the HADS for depression and anxiety each which is 'normal', that is, no depression/anxiety, comparative studies showed the following preoperative values: 82.0% (23/128) no anxiety and 79.8% (26/128) no depression [12], 64.8% (35/54) no anxiety and 77.8% (42/54) no depression [30], and finally 62.5% (25/40) with no anxiety or depression in a study that did not specify separate scores for HADS anxiety and depression [7].

Several studies, including the present study, show that TKA improves physical and mental well‐being, but the pathways by which TKA exerts such effects are unknown. Another possibility for mental health improvement is a substantial decrease in the state of chronic pain, which is a significant cause of anxiety and depression. This decrement can be followed by further enhancement in mental health [42]. Furthermore, improved mobility and greater postoperative functional independence can help restore postoperative patients' self‐efficacy and general well‐being [28]. Social and psychological influences, including increased participation in daily functioning and decreased dependence on caregivers, might also have an anxiolytic and antidepressant effect [8]. Additional research is warranted to delineate the exact biological and psychological mechanisms by which TKA will beneficially affect mental health.

Additionally, our results showed a strong relationship between age, depression, and anxiety. These results can be explained by the fact that growing older and developing health issues raise the risk of anxiety and despair [47]. Social ties are essential for everyone's survival and well‐being. However, people frequently discover that they spend more time alone as they age. Research indicates that there is a correlation between increased incidence of depression and social isolation and loneliness [44]. Also, we discovered a strong correlation between anxiety and depression, which is valid, given that anxiety may appear as a symptom of clinical (severe) depression. Anxiety disorders like separation anxiety disorder, panic disorder, or generalised anxiety disorder can also frequently cause depression. Many people are diagnosed with both clinical depression and anxiety disorders [19].

Most people maintain strong family bonds and social ties in the broader context of Jordanian society and culture. Given that knee OA is a painful and functionally limiting condition, finding the necessary home support to ease the physical deficits (walking difficulty) is a great mental relief. The social support comforts any depressive or anxiety symptoms the patients might experience and, in a way, reduces the negative modifier of social isolation/loneliness. This is especially significant for our sample, where 81% are above 60 years of age. This might explain any regional variation between our results and other international studies.

The OKS has greater reliability and validity than comparable measurement methods in terms of response rate and usability, and it is widely recognised as a valid and reliable measure of outcome following knee replacement [13, 16]. Since filling out the questionnaire and determining the levels of depression, anxiety, pain, and knee function is relative based on the person, there remains a kind of subjectivity in this research, and it is difficult to compare the results accurately with other research.

OKS improved significantly by 3 months after surgery; the mean for OKS was 17.9 ± 8.73 before the operation and increased to 38.4 ± 9.39 after the operation. On average, it increased by 20.5 points. These results concur with many previous studies, one of which was conducted in Italy; their results showed that the mean OKS before surgery was 20.2 and improved to 23.6 (*p* < 0.01) at 6 weeks post‐procedure [41]. An additional study involving 57 patients found that, on average, patients' OKS increased by 16.5 points (SD 8.5), indicating an improvement in knee function following TKA (*p* < 0.01). Before surgery, the mean OKS was 20.3 (SD 6.9), and following surgery, it was 36.8 (SD 6.8) [48]. Moreover, a mean 6‐month change of 14.5 was reported by Judge et al. [31]. and a change of 14.7 by Beard et al. [4]. This indicates a slightly greater shift in our patient population.

Another study done by Liru Ge et al. demonstrated that the TKA significantly reduced knee pain and function over a 60‐month period and improved physical health over a 48‐month period when compared to those with comparable degrees of knee osteoarthritis who did not have a TKA; however, this did not result in enhanced mental or physical activity levels [17].

Another important finding is that although patients with lower pre‐operative OKS scores improved more, their post‐operative results were still worse than those with higher pre‐operative scores. This aligns with multiple research findings [32, 64]. It might make sense that individuals with lower pre‐operative OKS scores would recover more from TKA. However, even then, their pain level remained higher than that of those with a higher pre‐operative score for pain [38, 60]. Patients with lower pre‐operative OKS may have had a greater shift in OKS scores since they have more ‘room’ to improve. The subjectivity and complex character of pain may help to explain this. Patients self‐rate their levels of sensation of pain and thresholds prior to and following surgery. Still, additional elements like their expectations and psychological aspects would influence how the scores are assigned [32, 67].

In our research, we did not find a statistically significant relationship between smoking and mental health outcomes, with a *p*‐value of 0.07. This suggests that, among patients undergoing total knee replacement, smoking is likely not a primary cause of anxiety and depression. Previous studies have indicated that smoking is generally associated with poorer mental health, showing higher rates of both anxiety and depression [27]. However, in patients undergoing major surgeries like total knee replacement, the stress of recovery and the physical limitations imposed by the surgery may overshadow the psychological effects typically linked to smoking.

Additionally, other psychological factors, such as coping strategies, pain management, and support systems, may play a more significant role in influencing mental health outcomes in this population compared to smoking alone. Patients recovering from surgery often focus on their physical rehabilitation, which may lead them to pay less attention to lifestyle factors like smoking. Further research is needed to explore whether the impact of smoking on mental health differs among surgical populations compared to the general population.

Furthermore, our research has revealed a significant inverse relationship between osteoporosis and mental health outcomes. Some findings suggest that higher levels of anxiety and depression are associated with more advanced osteoporosis. At first glance, this may seem confusing, as osteoporosis is typically correlated with increased anxiety and depression due to fears of fractures and mobility issues [34]. However, patients undergoing total knee replacement may have adapted to their chronic conditions, including osteoporosis, by concentrating on recovery and rehabilitation post‐surgery.

Other studies have indicated that chronic illnesses like osteoporosis can lead to a lower quality of life and increased psychological distress, largely due to fears of fractures and the limitations these conditions impose [41]. Nevertheless, the focus of care may shift towards joint recovery in patients after knee replacement, potentially alleviating some psychological burdens commonly associated with osteoporosis.

Caring for patients who undergo TKA places a significant financial strain on healthcare systems. This burden includes costs associated with the surgery, post‐operative care, rehabilitation and potential complications [40]. However, the long‐term benefits, such as enhanced mobility and improved quality of life compared to ongoing pain management or medication, can help to offset these costs [18].

A key question is whether the reduction in depression and anxiety following TKA leads to decreased use of healthcare resources. Many studies indicate that improved mental health after surgery results in a lower reliance on opioid prescriptions, fewer follow‐up visits, and reduced readmission rates [65]. Chronic pain combined with psychological distress often results in increased healthcare visits. Addressing these issues through TKA highlights the potential for significant clinical benefits [52]. However, further research is needed to quantify the financial savings associated with these outcomes accurately and to identify strategies that maximise patient results while minimising healthcare costs.

There are various strengths in this study. Since the reference population comes from the same country, there are fewer disparities in the environmental and psychosocial features between them. Furthermore, this study employs OKS, which is dependable and employed in the larger patient‐reported outcome measures (PROMs) programme in England and Wales. Without the help of clinicians, patients can finish OKS on their own. By doing this, bias reporting is eliminated. Additionally, because the OKS questions have been designed specifically for the knee joint, unrelated comorbidities impact the rating less. Finally, we used more than one scale in our investigation, which made this study cover multiple outcomes.

Our study is limited because the reference population's data was gathered voluntarily, making it susceptible to response bias. Second, our study may be limited by the brief follow‐up period following the operation. Finally, the fact that our investigation is centred on a particular site may restrict how broadly the study's conclusions may be applied. Moreover, selection bias may have occurred because all study participants were treated at the same hospital.

## CONCLUSION

Patients' quality of life may generally improve after total knee replacement surgery. It takes a few months following the treatment to notice these positive effects. Pain and function are the most important enhancement markers in an individual's life. Following a TKA, psychological demand (depression and anxiety) drops to an acceptable level. The overall OKS rating indicates a considerable improvement in pain and physical performance following TKA. Discussing psychological factors with patients preoperatively might be important along with the functional outcome in making the surgical decision.

## AUTHOR CONTRIBUTIONS

Bassem Hddad and Tasneem N. Alhosanie conceived the idea, designed the study, did data analysis, and performed writing—original draft and review. Bassem Haddad, Tasneem N. Alhosanie and Ali Kanaan designed the preliminary survey. Noor Yousef, Abdel Rahman Abuawad, Feras Abuhajleh and Mohammad Hamdan participated in original drafting and review. Ali Kanaan, Leen A. Mzahereh, Khuzama Mohammad, Aya Al‐Zurgan, Leen Alzghoul, Tala M. Mesmar and Fahad Alabhoul performed data collection. All authors read and approved the final manuscript.

## CONFLICT OF INTEREST STATEMENT

The authors declare no conflicts of interest.

## ETHICS STATEMENT

This study was reviewed and approved by the IRB of the University of Jordan Hospital. Every patient received an explanation of the main goal of the study as well as information regarding their ability to decline or withdraw from it. Additionally, throughout data analysis, patients were educated about anonymity and confidentiality.

## Data Availability

The data supporting the findings of this study are available from the corresponding authors upon request.
